# Hyperkalemia in chronic peritoneal dialysis patients

**DOI:** 10.1080/0886022X.2022.2032151

**Published:** 2022-02-15

**Authors:** Andrew B. Elliott, Karim M. M. Soliman, Michael E. Ullian

**Affiliations:** Division of Nephrology, Department of Medicine, Medical University of South Carolina, Charleston, SC, USA

**Keywords:** Peritoneal dialysis, hyperkalemia, hypokalemia

## Abstract

**Background**. Chronic peritoneal dialysis (PD) patients often develop hypokalemia but less commonly hyperkalemia.

**Methods**. We explored incidence and mechanisms of hyperkalemia in 779 serum samples from 33 patients on PD for 1 − 59 months. Normal serum potassium concentration was defined as 3.5 − 5.1 meq/l.

**Results**. Mean monthly serum potassium concentrations were normal (except for 1 month), but we observed hypokalemia (<3.5 meq/l) in 5% and hyperkalemia (>5.1 meq/l) in 14% of 779 serum samples. Incidence of hyperkalemia did not change over time on PD: Year 1 (15%), Year 2 (11%), Year 3 (19%), Years 4–5 (22%). Hyperkalemia was mostly modest but occasionally extreme [5.2–5.4 meq/l (55%), 5.5–5.7 meq/l (21%), 5.8–6.0 meq/l (10%), >6.0 meq/l (14%)]. Of 31 patients (2 excluded due to brief PD time), 39% displayed hyperkalemia only, 23% displayed hypokalemia only, and the remainder (38%) displayed both or neither. Comparing hypokalemia-only with hyperkalemia-only patients, we found no difference in potassium chloride therapy, medications interrupting the renin-angiotensin system, small-molecule transport status, and renal urea clearance. We compared biochemical parameters from the hypokalemic and hyperkalemic serum samples and observed lower bicarbonate concentrations, higher creatinine concentrations, and higher urea nitrogen concentrations in the hyperkalemic samples (*p* < 0.001 for each), without difference in glucose concentrations.

**Conclusion**. We observed hyperkalemia 3 times as frequently as hypokalemia in our PD population. High-potassium diet, PD noncompliance, increased muscle mass, potassium shifts, and/or the daytime period without PD might contribute to hyperkalemia.

## Introduction

Renal replacement options consist of hemodialysis, peritoneal dialysis (PD), and renal transplantation. In PD, the peritoneal lining acts as the semi-permeable membrane for solute removal. Advantages of PD include the ability of patients to perform their dialysis at home, hemodynamic stability during dialysis, ease of travel, and lack of needle use. Bacterial peritonitis and hypoalbuminemia are the major disadvantages of PD, whereas other less serious side effects can occur, including hernias, pneumoperitoneum, hemoperitoneum, and hypokalemia. Since peritoneal dialysate contains no potassium, hypokalemia is not unexpected in patients on chronic PD. The literature on PD supports this supposition [1–16], with less information about hyperkalemia. However, we have observed episodes of hyperkalemia, some extreme, in our PD population. Therefore, we explored the incidence, natural history, and potential mechanisms of hyperkalemia in our PD population. Dyskalemia in PD patients is clinically important, due to resulting increase in morbidity and mortality.

## Patients and methods

### Patient selection

In the Spring of 2018, we reviewed the charts of all patients, excluding those less than 19 years of age, on chronic PD in our home dialysis program in Charleston, South Carolina. This unit is managed by the nonprofit dialysis provider Dialysis Clinic Incorporated and is affiliated with the Division of Nephrology at the Medical University of South Carolina. This single-center, retrospective study was approved by the Institutional Review Boards of the Medical University of South Carolina and Dialysis Clinic Incorporated. The adult PD census at the time of chart review was 33 patients.

### PD prescription

Almost all patients were started on continuous manual PD after training, usually 4 exchanges of 2000 mL for 1 month, followed by peritoneal equilibration testing and then conversion to cycler PD at night (8 − 10 h) with a long ‘dry’ period (empty peritoneum) in the day. Over the next 6 − 12 months, the total daily inflow volume was increased as necessary to achieve a total urea clearance [KT/V (PD) + KT/V (urine)] of > 1.7/week. We opted for automated/intermittent PD (nightly PD with an empty peritoneal space in the day) in most patients because residual renal function was sufficiently maintained to achieve target total urea clearance. In addition, an empty peritoneum in the day obviated gastric compression and early satiety of appetite. Potassium chloride was never added to the PD solution bags but was prescribed orally in a few patients. No patients used salt substitutes.

### Blood and data collection

We reviewed the monthly routine laboratory concentrations (potassium, bicarbonate, creatinine, blood urea nitrogen, glucose concentrations) of the 33 patients, from the Spring of 2018 back to when they started PD. Month 1 was defined as the month after PD training. Serum chemistry values from Month 1 were censored, in case there was clinical instability in any of these new PD patients. We also documented age in 2018, ethnicity, sex, cause of renal failure, PD vintage (months on PD in 2018), chronic medications throughout the time on PD, most recent urinary urea clearance (KT/V urine), and 4-h dialysate/plasma creatinine ratio from the peritoneal equilibration test at the end of Month 1.

Since most patients dialyzed *via* PD cycler at night and had an empty peritoneum in the daytime, the time between the end of cycler PD (early morning) and blood collection (late morning or early afternoon) ranged from 3 to 7 h. After monthly phlebotomy, the blood tubes for chemistry values were centrifuged immediately for serum separation. Serum was sent by overnight mail to the laboratory of Dialysis Clinic Incorporated in Nashville TN. Serum chemistry values were measure with a Roche Cobas 502/701 analyzer. The normal range of serum potassium concentrations [K^+^]_s_ was defined as our hospital standard: 3.5 − 5.1 meq/l. Laboratory data were censored if the laboratory reported that blood samples were hemolyzed, but none were found to be so.

### Statistical considerations

Data are presented as mean and standard deviation. Group means were compared by 2-sided *t*-test, with significance at the *p* < 0.05 level. We studied the number of patients in our program when we began to investigate our question; power analysis to achieve sufficient number of patients was not performed because expected differences were unknown.

## Results

### Patient characteristics

Table 1 displays the demographic characteristics of the entire cohort at the time of the chart review in the Spring of 2018. Average age was 55.2 ± 16.5 years. Ethnicity was mostly African-American and White in virtually equal proportions with 2% Hispanic. Sex distribution was virtually equal as well. Renal failure was caused predominantly by diabetes mellitus, followed by focal segmental glomerulosclerosis, hypertension, systemic lupus erythematosus, kidney transplant failure, and other causes (nephrotoxic agent, IgA nephropathy, Alport Syndrome, obstructive nephropathy, sickle cell disease, scleroderma). PD vintage (time on PD) averaged just under 2 years.

**Table 1.** Entire cohort demographics.

Demographic	Value
Age (years)	55.2 ± 16.5
Sex (% Male/% Female)	48/52
PD vintage (months on PD)	23.3 ± 15.0
Ethnicity (%):	
African-American	52
White	46
Hispanic	2
Cause of kidney failure (%):	
Diabetic nephropathy	42
Focal segmental glomerulosclerosis	12
Hypertensive nephrosclerosis	9
Lupus nephritis	6
Failed transplant	6
Others*	25

*Nephrotoxins, IgA nephropathy, Alport, Obstruction, Sickle cell disease, Scleroderma.

### [K^+^]_s_ over time on PD

First, we assessed the effect of PD vintage (time on PD) on [K^+^]_s_. As mentioned above, we ignored laboratory values in the first month after training, assuming that the patients had attained clinical stability on PD by the second month. We expected that hyperkalemia might be more common in the early months of PD because patients might have been unsure of PD technique just after the completion of training and thus performed less PD than prescribed, and/or a small PD prescription might have been created just after training, since residual renal function persists early in the course of end-stage renal disease. However, mean monthly [K^+^]_s_ over Months 2 − 59 of PD were predominantly within the normal range without obvious trend over the months; only 1 mean monthly concentration was out of range: 5.22 ± 0.67 meq/l in the 40th month on PD (Table 2). The lowest mean [K^+^]_s_ was 4.00 ± 0.35 meq/l in the 50^th^ month on PD. Mean [K^+^]_s_ were not included in Table 2 for months 52 − 59, since there were less than 3 patients on PD for more than 51 months.

**Table 2.** Mean [K^+^]_s_ over time on PD.

Month on PD	Serum K+ (meq/l)	Month on PD	Serum K+ (meq/l)	Month on PD	Serum K+ (meq/l)
1	NR	21	4.51 ± 0.51	41	4.90 ± 0.84
2	4.72 ± 0.65	22	4.42 ± 0.68	42	4.55 ± 0.57
3	4.43 ± 0.87	23	4.16 ± 0.58	43	4.80 ± 0.96
4	4.41 ± 0.72	24	4.31 ± 1.04	44	4.63 ± 0.80
5	4.50 ± 0.51	25	4.53 ± 0.59	45	4.46 ± 0.66
6	4.52 ± 0.56	26	4.36 ± 0.52	46	4.67 ± 0.75
7	4.50 ± 0.73	27	4.42 ± 0.61	47	4.37 ± 0.05
8	4.33 ± 0.59	28	4.37 ± 0.72	48	4.63 ± 0.70
9	4.62 ± 0.59	29	4.32 ± 0.47	49	4.57 ± 0.72
10	4.46 ± 0.78	30	4.44 ± 0.57	50	4.00 ± 0.35
11	4.29 ± 0.67	31	4.79 ± 0.56	51	5.03 ± 1.46
12	4.42 ± 0.59	32	4.48 ± 0.57		
13	4.42 ± 0.74	33	4.36 ± 0.68		
14	4.38 ± 0.69	34	4.61 ± 0.51		
15	4.31 ± 0.80	35	4.54 ± 0.54		
16	4.26 ± 0.59	36	4.49 ± 0.63		
17	4.17 ± 0.60	37	4.65 ± 0.47		
18	4.35 ± 0.60	38	4.63 ± 0.58		
19	4.44 ± 0.58	39	4.47 ± 0.31		
20	4.57 ± 0.82	40	5.22 ± 0.67		

Data from Months 52–59 were not included, due to small numbers. NR: not reported.

**Figure 1.**
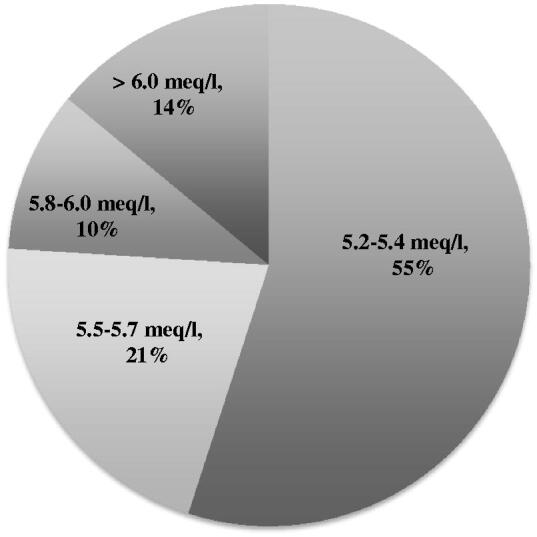
Severity of hyperkalemia. This pie chart demonstrates the severity of hyperkalemia in the 110 hyperkalemic serum samples out of a total of 779 serum samples, with severity of hyperkalemia graded as follows: 5.2–5.4 meq/l, 5.5–5.7 meq/l, 5.8–6.0 meq/l, and >6.0 meq/l. The 110 hyperkalemic values represent 100%. Severe hyperkalemic values (>6.0 meq/l) were 6.1, 6.2, 6.3, 6.3, 6.4, 6.4, 6.7, 6.7, 6.8, 6.9, 7.0, 7.9 meq/l.

**Figure 2.**
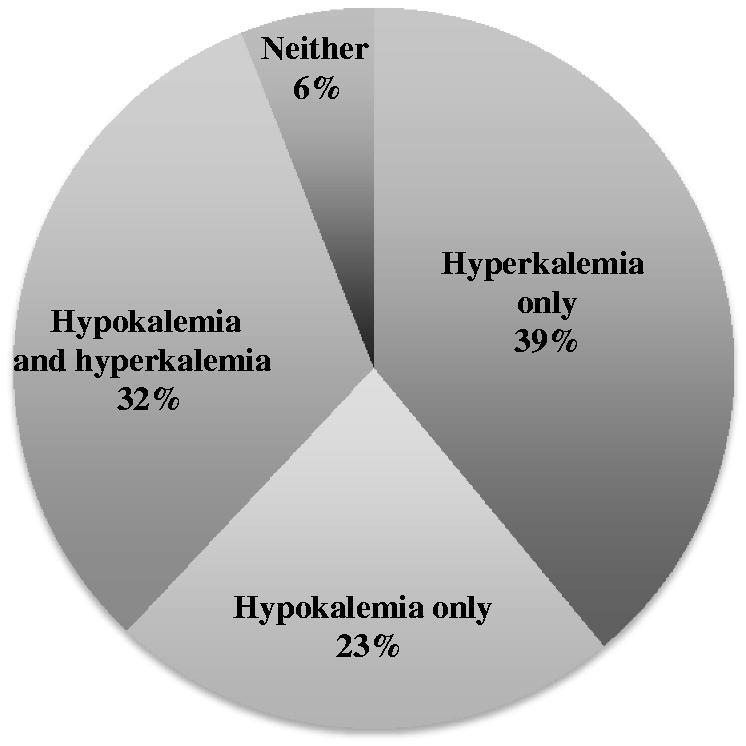
Hyperkalemia and/or hypokalemia in the patient population. This pie chart delineates which of the 31 patients (2 were excluded due to being on PD for only 2 months) were: hyperkalemic only, hypokalemic only, never hyperkalemic or hypokalemic, or sometimes hyperkalemic and other times hypokalemic. The 31 patients represent 100%.

When we assessed each of the 779 monthly chemistry values over the course of 59 months in the 33 patients, we observed hypokalemia (<3.5 meq/l) in 40 samples (5%) and hyperkalemia (>5.1 meq/l) in 110 samples (14%). Thus, hyperkalemia was found almost 3-fold more frequently than hypokalemia. The incidence of hyperkalemia did not differ appreciably in each year after the initiation of PD, that is, with time on PD: Year 1 (15%), Year 2 (11%), Year 3 (19%), and Years 4/5 (22%); values from Years 4 and 5 were combined because the number of values in those years were small. Hyperkalemia was predominantly modest, with about 50% of hyperkalemic values between 5.2–5.4 meq/l, and severe hyperkalemia (>6.0 meq/l) occurred in 14% of hyperkalemic values (Figure 1). Of 31 patients (2 were not included because laboratory values were available for 1 month only, i.e. in the second month on PD), 39% (12 patients) developed hyperkalemia only, 23% (7 patients) developed hypokalemia only, 6% (2 patients) were never hyperkalemic or hypokalemic, and 32% (10 patients) developed episodes of hyperkalemia and hypokalemia (Figure 2).

### Potential mechanisms of hyperkalemia

None of the hyperkalemic samples were reported to be from a hemolyzed blood sample. We examined clinical and biochemical data to try to explain the mechanisms by which patients became hyperkalemic. Using the 7 hypokalemia-only patients as a control group for the 12 hyperkalemia-only patients, we found no statistically significant differences between the groups in the use of potassium chloride therapy (3 patients only in the entire population), medications that interrupt the renin-angiotensin system (43% vs. 17%, respectively), small-molecule transport status (71% vs 67% low/low-average transporters, respectively), or residual renal urea clearance, i.e. KT/V from urine: 0.44 ± 0.50 vs 0.50 ± 0.63 per week, respectively.

We then compared biochemical parameters from the 40 hypokalemic serum sample subset with those from the 110 hyperkalemic serum sample subset (of the total 779 samples), and we observed lower bicarbonate concentrations, higher creatinine concentrations, and higher urea nitrogen concentrations in the hyperkalemic samples (*p* < 0.01 for each), without difference in glucose concentrations (Table 3). Since higher serum creatinine concentrations might be consistent with higher muscle mass in younger patients as a mechanism for hyperkalemia, we revisited the 7 hypokalemia-only patients and the 12 hyperkalemia-only patients and compared their ages. We found no difference in ages (mean ± SD) between these patient groups: 53.3 ± 16.9 versus 54.2 ± 16.5, respectively, *p*-value 0.45 (NS).

**Table 3.** Biochemical parameters in hypokalemic and hyperkalemic serum samples.

Serum biochemical parameter	Hypokalemic serum samples	Hyperkalemic serum samples	*p* Value
Number of samples	40	110	–
Bicarbonate (22–29 meq/l)	24.2 ± 2.4	22.4 ± 3.5	<0.01
Glucose (70–100 mg/dl)	130 ± 45	128 ± 52	NS
Creatinine (0.7–1.3 mg/dl)	9.9 ± 4.0	12.6 ± 5.5	<0.01
BUN (8–26 mg/dl)	49 ± 12	61 ± 18	<0.01

BUN: blood urea nitrogen; normal ranges and units are included within the parentheses.

## Discussion

### Recapitulation of our findings

This retrospective chart review of [K^+^]_s_ in PD patients from a single home dialysis unit demonstrated that hyperkalemia, sometimes severe, was much (3-fold) more common than hypokalemia, with the normal range of [K^+^]_s_ adopted from the hospital standard of 3.5 − 5.1 meq/l. The incidence of hyperkalemia did not change with time on PD, which ranged from 1 month to almost 6 years. We found no relationship between hyperkalemia and any of the following: potassium chloride supplements, angiotensin converting enzyme inhibitors or angiotensin receptor blockers, small-molecule transport status, or renal urea clearance. Hyperkalemic (compared to hypokalemic) serum samples were characterized by lower bicarbonate concentrations, higher creatinine concentrations, and higher urea nitrogen concentrations but not hyperglycemia.

### [K^±^]_s_ in chronic PD patients: review of the literature

Most of the reports from the medical literature on [K^+^]_s_ in chronic PD patients mention hypokalemia (rather than hyperkalemia), and some of these manuscripts include comparisons of hemodialysis and PD populations, which demonstrate higher [K^+^]_s_ in the former and lower [K^+^]_s_ in the latter [1–16]. Several publications report hypokalemia but do not mention the presence or absence of hyperkalemia [7,9,15,16]; it is possible that hyperkalemia was present in the PD population of these 4 studies but not reported, since hypokalemia was the thrust of the research.

Some investigators have reported hyperkalemia in chronic PD patients, although these reports are not as common as the reports of hypokalemia. Of 82 patients starting PD with normal [K^+^]_s_, 7.3% developed hypokalemia and 10% developed hyperkalemia after 12 months [12]. In 84 patients on chronic PD for 3 months, 14.3% developed hypokalemia and the same percent developed hyperkalemia [13]. In a larger study (319 incident PD patients), [K^+^]_s_ was assessed during the first 3 months of dialysis, and hypokalemia was observed in 4% and hyperkalemia was observed in 17% [17]. In 68 patients on PD for at least 6 months, 10% developed hypokalemia and 16% developed hyperkalemia [10]. Rarely, severe hyperkalemia (> 6.0 meq/l) in chronic PD patients has been reported [4,5,18–20].

### Mechanism of hyperkalemia in chronic PD patients

It is not surprising that hypokalemia is reported in chronic PD patients, since the chemical gradient for potassium from blood to peritoneal dialysate, which contains no potassium, is large. It is more difficult, however, to understand why chronic PD patients develop hyperkalemia.

In several case reports of severe hyperkalemia in PD patients, the etiology was considered to be high potassium intake, such as salt substitutes [18] and potassium-containing weight loss regimens [19]. It is possible in these cases that the large potassium intake overwhelmed diffusion down the chemical gradient from blood to dialysate. Due to commonly described occurrences of hypokalemia in chronic PD patients, the home program staff (nephrologists, nurses, dieticians) often urge PD patient to ingest a high-potassium diet. These diets, like the salt substitutes and potassium-containing weight loss regimens mentioned above, might result in positive potassium balance and hyperkalemia.

It is well known that [K^+^]_s_ is lowest just after the completion of hemodialysis and highest at the beginning of the next hemodialysis. Similarly, in patients dialyzing only at night on the cycler with an empty peritoneum in the day, one might consider that hyperkalemia could have developed if there was a large lag between the time that PD concludes and the time that blood samples were obtained later in the day. The time between the end of cycler PD (early morning) and blood collection (late morning or early afternoon) ranged from 3 to 7 h, which is far less than the time from the end of a hemodialysis session to the beginning of the next session (when the monthly blood samples were obtained), about 44 h. Therefore, it is unlikely that hyperkalemia occurred due to tissue release or enteral intake of potassium during the time period from the end of PD and to blood collection. We found nothing about this in the PD literature.

Another case report described hyperkalemia in a chronic PD patient with hepatitis, which resolved when the hepatitis resolved; the mechanism was interpreted as redistribution of potassium from injured liver tissue to the circulation [20]. We found that hyperkalemic serum samples also contained lower bicarbonate concentrations, that is, presumed metabolic acidosis (Table 3). This suggests that hyperkalemia was caused by exchange of protons (extracellular to intracellular) for potassium (intracellular to extracellular), as a mechanism to correct the acidosis.

We found that serum samples with higher [K^+^]_s_ also contained higher creatinine concentrations and urea nitrogen concentrations. Similar to our findings were those from Liu et al., who observed a positive correlation between azotemia (creatinine and urea nitrogen levels) and [K^+^]_s_ [17]. It is possible that noncompliance with PD therapy, which would be reflected by more azotemia, caused hyperkalemia. It is also possible that larger muscle mass or greater protein intake contributed to hyperkalemia. It has been demonstrated that skeletal muscle content of potassium is greater in PD patients than in control patients with normal renal function [21]. A carefully performed metabolic study in 8 chronic PD patients demonstrated that increasing daily dietary protein intake from 0.98 to 1.44 g/kg resulted in positive nitrogen balance and positive potassium balance [22]. We did not find that hyperkalemia-only patients were younger than hypokalemia-only patients, with youth a putative marker of larger muscle mass.

Although we did not find an association between medications that interrupt the renin-angiotensin-aldosterone system and hyperkalemia, this association has been investigated by others. In a prospective study of 21 normokalemic PD patients, treatment for 4 weeks with an angiotensin converting enzyme inhibitor or angiotensin receptor blocker did not change [K^+^]_s_ from baseline [23]. Similarly, in an observational study of 636 stable PD patients, use of angiotensin converting enzyme inhibitors or angiotensin receptor blockers was not associated with higher [K^+^]_s_ [24]. In contrast, higher [K^+^]_s_ were associated with use of angiotensin converting enzyme inhibitors in a cross-sectional study of 32 PD patients, independent of residual renal function, and the authors postulated that angiotensin converting enzyme inhibitors limited the movement of potassium from the blood to the peritoneal fluid [11].

### Limitations of our study

The small number of patients is a limitation of our study, as is the smaller number in the sub-groups, for example, ‘hypokalemia only’ and ‘hyperkalemia only.’ However, the total number of serum samples (> 750) obtained from the 33 patients is not small. Thus, the strength of this study is the large number of serum samples and the longitudinal nature of the study.

### Summary

This topic is clinically relevant, since mortality in chronic PD patients was reported to be 50% greater with hypokalemia (< 3.5 meq/l) or hyperkalemia (> 5.5 meq/l) compared to the normal range of [K^+^]_s_, between 3.5 and 5.5 meq/l [14]. In addition, variability of [K^+^]_s_ in chronic PD patients also results in increased in cardiovascular mortality [25]. We observed [K^+^]_s_ variability in 32% of our patients (Figure 2), who were hypokalemic in some months and hyperkalemic in others. Therefore, outcomes should be better if [K^+^]_s_ is maintained in the normal range. Based upon our results and those from the literature mentioned above, maneuvers to prevent or reverse hyperkalemia might include: (1) ensuring that all prescribed PD is performed by the patients, which could be facilitated by newer PD cyclers that send PD performance to the Home Program telemetrically; (2) adding a daytime dwell(s) to nighttime cycles so that there will be less time with an empty peritoneum for hyperkalemia to develop: (3) following closely the [K^+^]_s_ if a high-potassium diet is prescribed to avoid the common onset of hypokalemia; and (4) adding oral sodium bicarbonate therapy to prevent or correct metabolic acidosis, transfer of potassium from the intracellular space to the serum, and resulting hyperkalemia.
